# Air–liquid interface culture combined with differentiation factors reproducing intestinal cell structure formation *in vitro*

**DOI:** 10.1242/bio.061612

**Published:** 2025-01-20

**Authors:** Isamu Ogawa, Takaaki Nakai, Takahiro Iwao, Tamihide Matsunaga

**Affiliations:** ^1^ Department of Clinical Pharmacy, Graduate School of Pharmaceutical Sciences, Nagoya City University, 3-1 Tanabe-dori, Mizuho-ku, Nagoya 467-8603, Japan; ^2^ Department of Molecular and Cellular Health Sciences, Graduate School of Pharmaceutical Sciences, Nagoya City University, 3-1 Tanabe-dori, Mizuho-ku, Nagoya 467-8603, Japan

**Keywords:** iPS cell, Intestinal organoid, Crypt-villus-like structure, Air–liquid interface culture

## Abstract

Reproducing intestinal cells *in vitro* is important in pharmaceutical research and drug development. Caco-2 cells and human iPS cell-derived intestinal epithelial cells are widely used, but few evaluation systems can mimic the complex crypt-villus-like structure. We attempted to generate intestinal cells mimicking the three-dimensional structure from human iPS cells. After inducing the differentiation of iPS cells into intestinal organoids, these were dispersed into single cells and cultured two-dimensionally. An air–liquid interface culture was used, with CHIR99021, forskolin, and A-83-01 used as key compounds. Long-term culture was also performed by adding Wnt3a, Noggin, and RSPO1, which are frequently used in organoid culture. The air–liquid interface culture combined several compounds that successfully induced the formation of a crypt-villus-like structure, which grew rapidly at around day 6. The expression of pharmacokinetic genes such as *CYP3A4* was also enhanced. The intestinal stem cells were efficiently maintained by the addition of Wnt3a, Noggin, and RSPO1. We were able to construct a crypt-villus-like structure on cell culture inserts, which is considered a very simple culture platform. This structure had characteristics extremely similar to living intestinal tissues and may have a superior homeostatic mechanism.

## Teaser

**Summary:** Physiological functions of intestinal cells can be reproduced, but their complex crypt-villus-like structure is difficult to mimic. A combination of air–liquid interface culture and differentiation-promoting factors makes it possible.

## INTRODUCTION

Evaluating the gastrointestinal absorption processes is vital in drug development, since oral drugs comprise a large proportion of pharmaceuticals. Caco-2 cells, a cell line derived from human colon cancer, are the gold standard in nonclinical studies, but using this has numerous challenges, including different transporter expression patterns and significantly lower cytochrome P450 (CYP) activity than in the human gastrointestinal tract (Harwood et al., 2016; Sun et al., 2002). Meanwhile, pharmacokinetic evaluation using laboratory animals is a relatively accurate way to mimic human conditions *in vivo,* but its caveats include animal ethics issues and the species difference from humans. This has prompted replacement with alternative methods that do not use animals. To address these problems, various attempts have been made to develop standards for novel intestinal evaluation systems, although none have been perfected yet.

Human induced pluripotent stem (iPS) cells have been recently established (Takahashi et al., 2007), resulting in the rapid development of research related to the human body (Iwao et al., 2014; Kodama et al., 2016; Kabeya et al., 2018; Iwao et al., 2015; Ogaki et al., 2013; Negoro et al., 2018; Onozato et al., 2021 et al, 2020 et al, 2018). Techniques for inducing differentiation into various organs have been developed, with several methods for the intestinal tract specifically. There are two main types of culture techniques for intestinal cells: general planar culture and three-dimensional (3D) culture, used form 3D structures known as intestinal organoids. We have previously investigated human iPS cell-derived intestinal organoids (HIOs) and reported a method for culturing HIOs with a budding-like structure and pharmacokinetic functions from human iPS cells (Onozato et al., 2021). HIOs are characterized by the abundance of multiple types of cells that constitute the intestinal tract (Spence et al., 2011; Uchida et al., 2017; Finkbeiner and Spence, 2013), which might be more versatile than two-dimensional (2D) structures.

Despite their excellent cellular properties, HIOs are not suitable to be used as an evaluation system for drug discovery research, such as in pharmacokinetic studies. HIOs have a hollow spherical structure, meaning that only their front or back can be exposed to the culture medium, which makes it difficult to observe how compounds such as drugs permeate the intestinal membrane. Additionally, HIOs vary in size and maturity, which can complicate an analysis. Thus, we explored a method to produce intestinal cells that can form 3D structures on commonly used culture plates. This study explores use of the air–liquid interface (ALI) culture of intestinal organoids to promote the self-assembly of intestinal cells to form 3D structures. Although several methods for producing 3D intestinal cells have been reported, most methods use microdevices or biogenic intestinal tissues (Kim et al., 2016; Stroulios et al., 2021). Notably, iPS cell-derived intestinal cells are difficult to control, and the formation of 3D structures on common culture vessels such as cell culture inserts has not been reported. The intestinal tract-specific cell turnover and the long-term culture made possible by this turnover would potentially be of great significance in studies of the intestinal tract. Furthermore, it would be very easy to use a planar culture on cell culture inserts with the apical and basal membrane sides both open. In this study, we developed a method to easily produce 3D intestinal cells from HIOs.

## RESULTS

### Young HIOs remain in good condition after 2D expansion

**Fig. 1.**
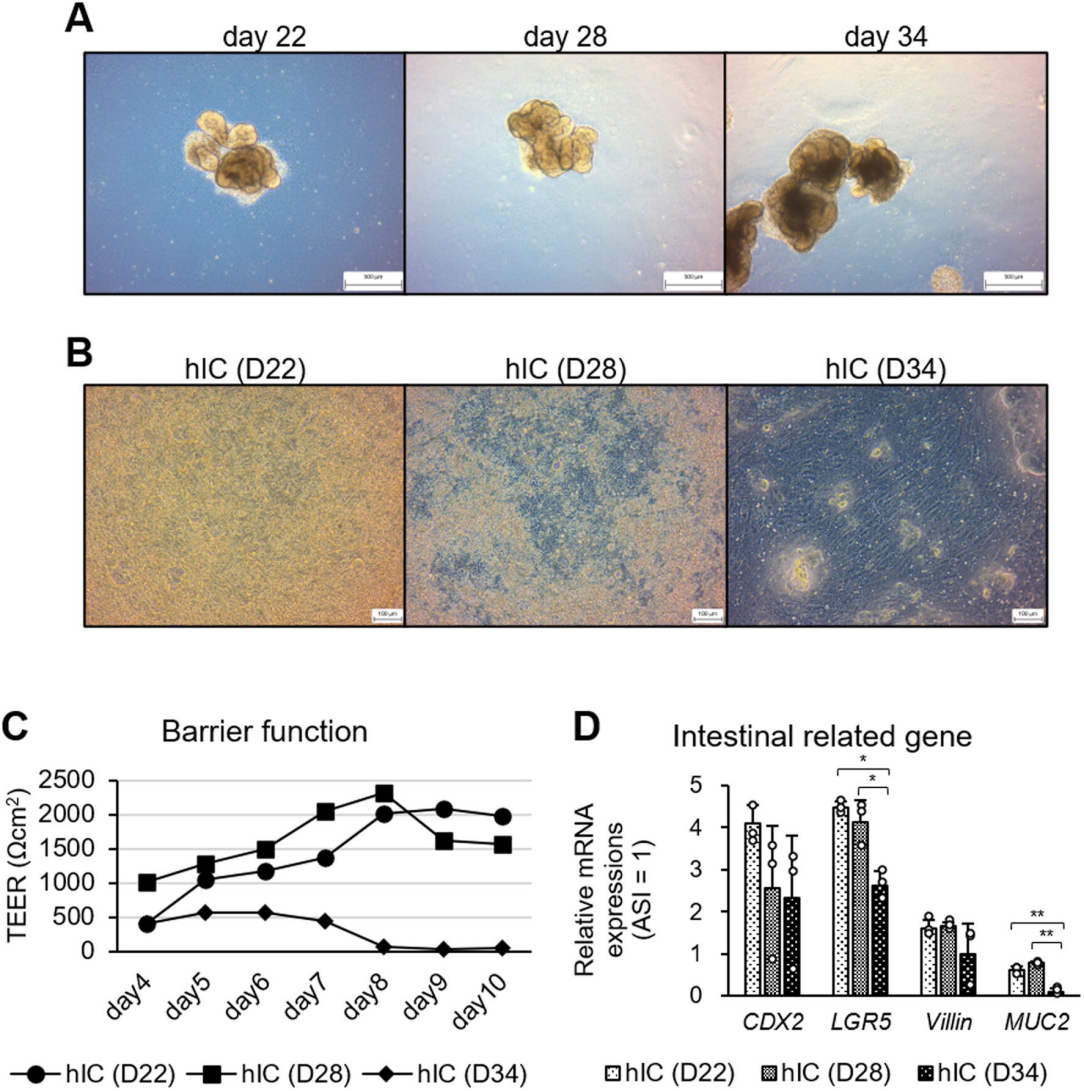
**Single-celled HIOs cultured in cell culture inserts.** (A) HIOs were generated via 3D culture. Days 22, 28, and 34 represent the number of days of induction of differentiation from iPS cells. Scale bars: 500 μm. (B) HIOs were dispersed into single cells and then subject to 2D culture in cell culture inserts for 10 days. Scale bars: 100 μm. (C) TEER was measured every day after 4 days of 2D culture. *n*=3. (D) mRNA expression levels for intestinal-related genes were measured by qPCR. The delta–delta method was used, and the data were normalized by HPRT. The relative values obtained from the adult small intestine (ASI) as 1 are shown. White dots: individual values; bars: averages. Mean±s.d. *n*=3. ^∗^*P*<0.05, ^∗∗^*P*<0.01.

HIOs cultured according to the protocol established in our laboratory (Onozato et al., 2021) were dispersed into single cells and expanded to the cell culture insert on different culture days. The HIOs formed sufficiently on day 22 (Fig. 1A), and the cells at 10 days after 2D expansion were more numerous in the 22- and 28-day HIOs (Fig. 1B). In single-cell formation, large clumps are removed using a detachment solution and a cell strainer. However, the 34-day HIOs exhibited strong cell adhesion, making single-cell formation difficult and adversely affecting cell proliferation after 2D expansion (Fig. 1B). The human iPS cell-derived intestinal-like cells (hICs) demonstrated high and stable trans-epithelial electrical resistance (TEER), an index of the intestinal barrier, specifically among hICs plated with detached 22- and 28-day HIOs (Fig. 1C), along with high mRNA expression of intestinal-related genes (Fig. 1D). In particular, the expression of *LGR5*, an intestinal stem cell marker, was higher on days 22 and 28 than on day 34. The hICs used in subsequent experiments were dispersed to single cells between days 22 and 28.

### ALI culture creates a 3D cell structure

**Fig. 2.**
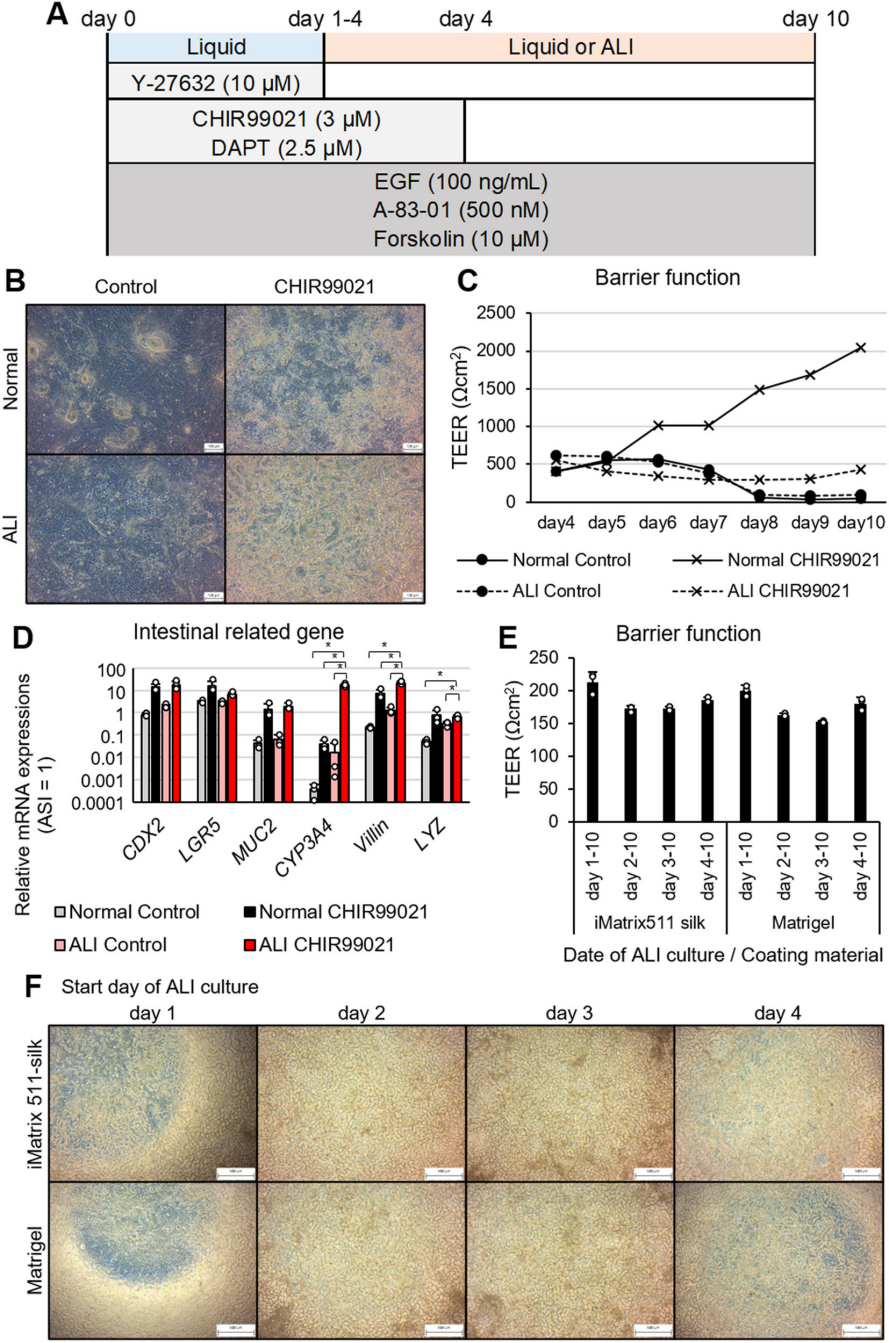
**3D structures formed by hICs in ALI culture and CHIR99021.** (A) A simple diagram of the compounds used and the timing of their use in the 2D culture protocol for hICs. (B) Photographs of single-celled HIOs cultured on cell culture inserts for 10 days. Normal: normal liquid-phase culture, control: no CHIR99021 added. ALI culture was started by removing the medium from the top of the insert on day 1. Scale bars: 100 μm. (C) TEER was measured every day after 4 days of 2D culture. For the ALI culture, the warmed medium was added to the insert only at the time of measurement. *n*=3. (D) mRNA expression levels for intestinal-related genes were measured via qPCR. The relative values obtained from the adult small intestine (ASI) as 1 are shown. White dots: individual values; bars: mean±s.d. *n*=3. ^∗^*P*<0.05. (E,F) ALI cultures were started on days 1, 2, 3 and 4. Cultures were incubated for a total of 10 days. The coating of the cell culture inserts was also compared between Matrigel and iMatrix-511 silk. Analysis was done on day 10 of incubation. TEER was measured 10 days after 2D culture. White dots: individual values; bars: mean±s.d. *n*=3. Scale bars: 500 μm.

**Fig. 3.**
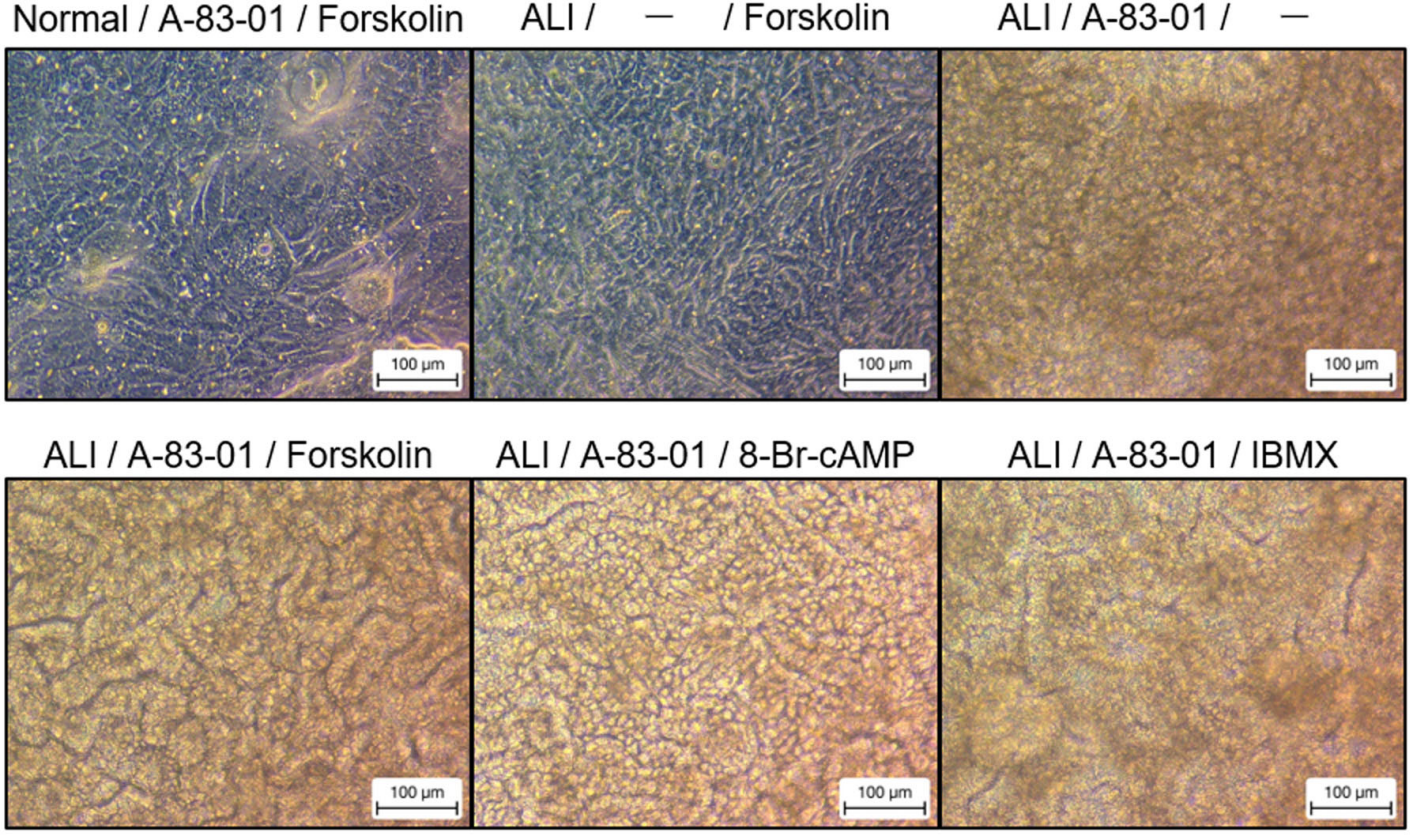
**Role of cAMP signaling and TGF-β signaling in the intestinal conformation.** The culture period was 10 days, and ALI culture was performed from days 4 to 10. A-83-01: 500 nM, forskolin: 30 µM, 8-Br-cAMP: 1 mM, IBMX: 500 µM. Scale bars: 100 μm.

ALI culture was performed in a 2D culture using the factors depicted in Fig. 2A. The combination of ALI culture and CHIR99021 turned the cells yellow in appearance. Micrographs suggested that thicker areas were discoloured (turned yellow) by light reflection and transmission, suggesting increased cell density and cell thickness (Fig. 2B). the total amount of mRNA obtained for RT-qPCR also confirmed that cell mass was higher in yellow looking cells (data not shown). The TEER was stable at approximately 300–500-Ω cm^2^ only in the group with ALI culture and CHIR99021 (Fig. 2C). Despite variations in data and the limited significant differences, the expression of intestinal-related genes was also increased by the combination of ALI culture and CHIR99021. Notably, there were synergistically increased expressions of the metabolic enzyme CYP3A4 and the intestinal epithelial cell marker villin (Fig. 2D). Meanwhile, the goblet cell marker MUC2 also showed an increasing trend, but it was not statistically significant. The duration of the ALI culture and the coating agent were then examined. The ALI culture was started on any day between days 1 and 4. There were no differences when comparing iMatrix-511 silk, which is commonly used for intestinal epithelial culture, versus Matrigel, which is used for organoid culture (Fig. 2E). When the ALI culture was initiated on day 2 or 3 of the culture, crypt-villus-like structures were formed over the entire insert (Fig. 2F). The shade of colour in the micrograph indicates the height of the cell. The black lines are groove-like concave structures (i.e. crypts), whereas the light-coloured areas are convex (i.e. villi). TEER was lower in cells with crypt-villus-like structures. When using hICs, we attempted to cryopreserve cells to shorten the time and improve the convenience of inducing differentiation from iPS cells. After investigating five commercially available cell preservation solutions, there were no abnormalities in cell morphology, cell viability, or gene expression, and thus all five solutions could be used without any problems (Fig. S1A-C). In addition to CHIR99021, forskolin and A-83-01 were used in this culture. To confirm the agents that contribute to the formation of the crypt-villus-like structure, we attempted to generate the crypt-villus-like structure under various conditions. The crypt-villus-like structure was not detected when forskolin was removed, we assumed that cyclic adenosine monophosphate (cAMP) signalling significantly contributed toward the its formation and acted as a substitute for other cAMP signal activators in the culture medium. Meanwhile, crypt-villus-like structures formed in the 8-Br-cAMP- and IBMX-supplemented groups, although with a slightly different morphology (Fig. 3). Furthermore, when comparing the hICs with (left side of the lower row) and without (middle of the upper row) A-83-01, the crypt-villus-like structure clearly disappeared in the latter. Nevertheless, the morphology can still be further improved by optimizing the concentration of the factors.

### ALI culture caused the formation of concave–convex structures and mucus production

**Fig. 4.**
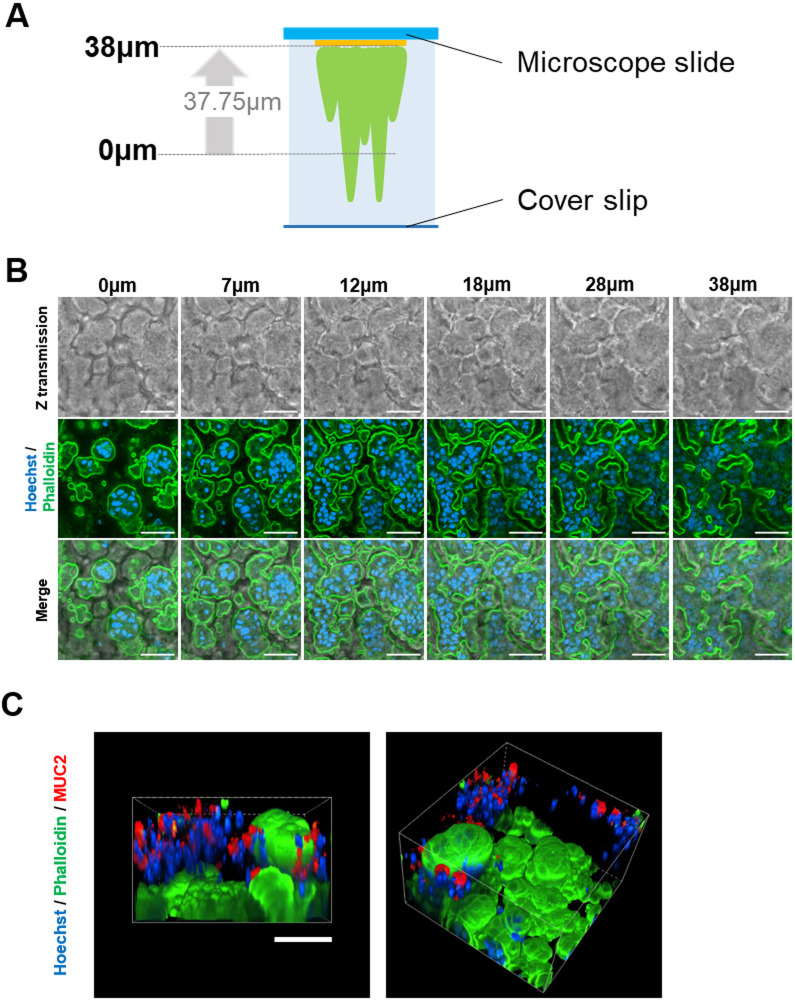
**Concave–convex 3D structure of completed hICs with microvilli and goblet cells.** (A) Cultured hICs were stained, cell culture inserts were cut out, and glass slides were prepared. The culture period was 10 days, and ALI culture with CHIR99021, A-83-01, and forskolin was performed from days 4–10. (B) Transmission and fluorescence images were acquired at a height of 38 µm from 0 µm in the Z-axis. The nuclei and microvilli were stained using Hoechst 33342 and phalloidin, respectively. Merged images are also shown. Scale bars: 50 μm. (C) Goblet cells and secreted mucus were stained with MUC2. Scale bars: 50 μm.

We produced hICs in ALI culture with CHIR99021, A-83-01 and forskolin, which is the best among the culture methods shown so far. Microscope slides were prepared and subject to immunofluorescence staining on day 10 of culture (Fig. 4A). Nuclei were stained blue using Hoechst 33342, while the microvilli were stained green using phalloidin. The microvilli were expressed in the contours of the convex area. The structure was 3D with concave and convex areas, indicating a cryptic villus-like structure (Fig. 4B, Movie 1). When the transmission image was merged in the Z-axis direction, it suggested that the dark areas were concave areas with no cells. Additionally, MUC2 staining suggested that mucin was secreted in the upper part of the cell (Fig. 4C).

### ALI culture produces a dense crypt-villus-like structure

**Fig. 5.**
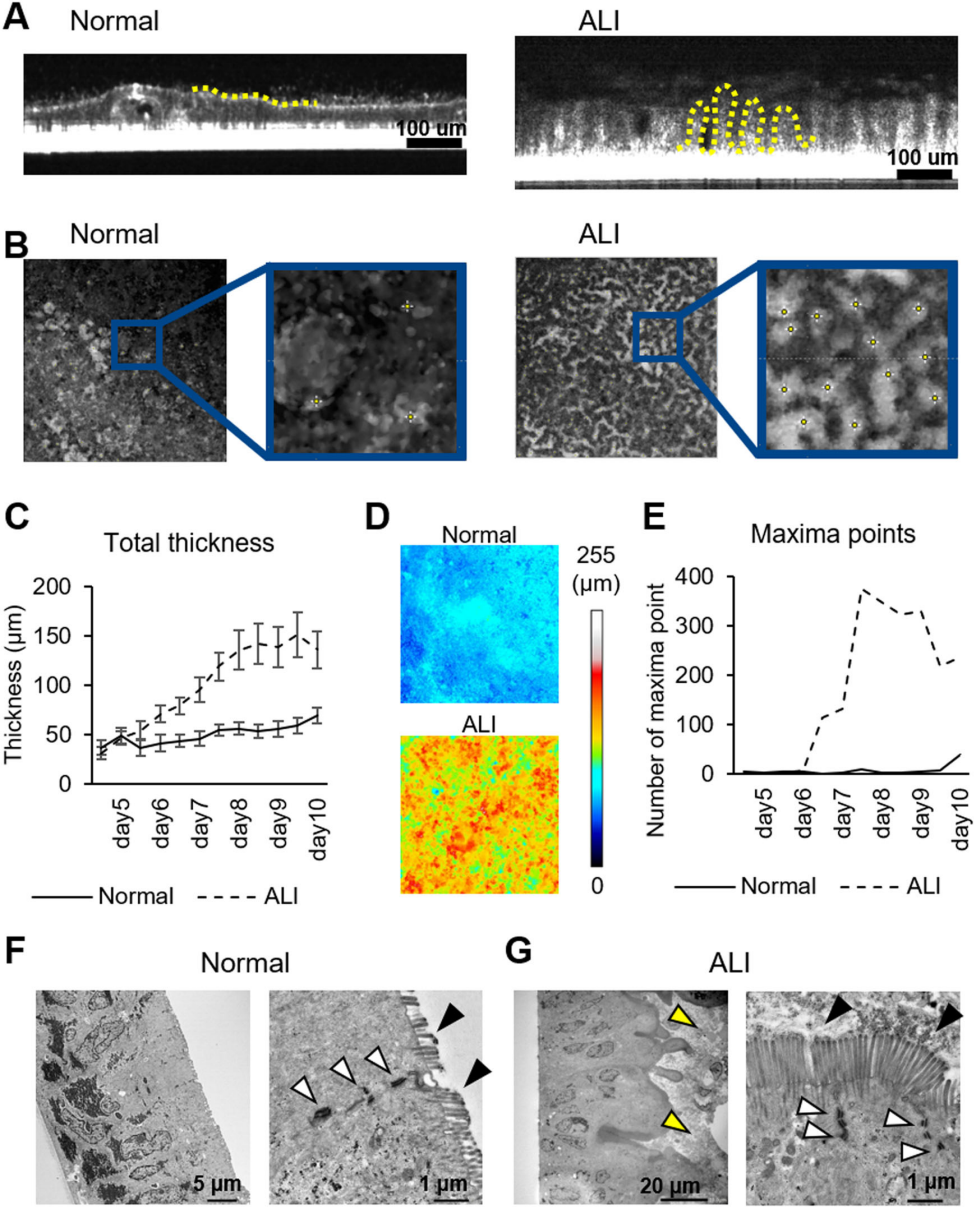
**Rapid growth of the crypt-villus-like structure from day 6 in culture.** (A) Cells were imaged over time using Cell3iMager Estier, and the images were processed using ImageJ. The figure shows a cross section. Yellow dotted line: apical surface. Scale bars: 100 μm. (B) The Z-section image was projected onto the Z-plane. White areas: convex areas; areas in blue: enlarged images; yellow dots: maxima points. (C) Cell height was recorded every 12 h from days 5–10. Values are averages within plots. (D) The cell height at day 10 is represented in the heat map. (E) The number of maxima was counted over time. *n*=1. (F,G) Transmission electron microscopy images of the normal and ALI culture conditions, respectively. The left image is at low magnification, and the right image is at high magnification. White arrows: tight junctions; black arrows: microvilli; yellow arrows: mucus-like material.

Cell3iMager Estier can detect the 3D structure of cells in culture using light interference. In the cross-sectional view, the cells and insert membranes are shown in white, while in the overhead view, the whiteness represents the thickness. On day 10, the cell surface was flat in the normal culture (shown by the yellow dotted line), whereas in the ALI culture, a crypt-villus-like structure was clearly observed (Fig. 5A,B). The cell sheet thickness increased with the number of days of culture in ALI (Fig. 5C,D, Figs S2 and S3). After counting the peak of each convexity as the ‘maximum point’, the convexity structure was rapidly generated at around day 6 (Fig. 5E, Fig. S4). The approximate number of convexities per area reached 350 on days 7–8 and remained at 300 until day 10, which explains the large thickness error of the cell sheets in the ALI culture. In contrast, the number of irregularities did not increase in the normal culture. The thickness and convexity of the cell sheet remained stable after day 8 of incubation, suggesting that the cell sheet was structurally complete. The convexity count decreased slightly on day 10 of culture, which was attributed to the accumulation of dead cells on the cell surface; this made it difficult to observe the concave-convex structure.

### ALI culture develops microvilli

The structure of the microvilli was further observed via transmission electron microscopy (TEM), which revealed that the apical membrane side had a linear structure in normal culture. Conversely, a concave and convex structure was noted in the ALI culture, and mucus-like material accumulated in the upper part of the cell (shown by the yellow arrows in Fig. 5G). This material is suggested to contain the MUC2 observed in Fig. 4C, and it is also clearly viscous, as it could not be washed away by the cell surface washing process while preparing the observation sample. Tight junctions were formed between cells, similar to the normal culture, and the microvilli were >1 μm in length (Fig. 5F,G). However, there was clearly a higher density of microvilli compared to the normal culture.

### Long-term culture is possible by adding maintenance culture factors

**Fig. 6.**
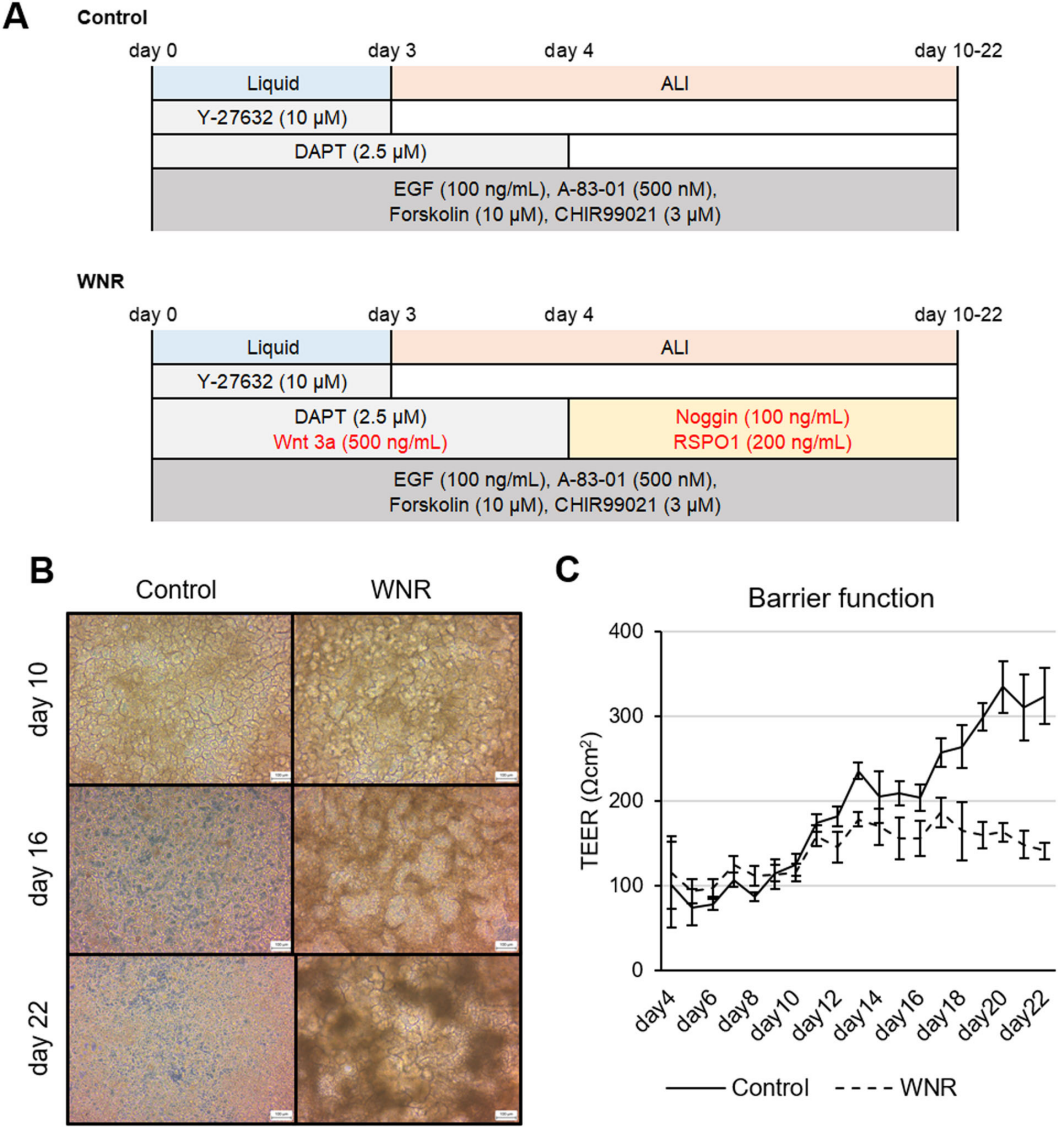
**3D structure maintained by the activation of intestinal stem cells.** (A) The 22-day long-term culture protocol for hICs in ALI culture is shown. Red letters: newly added compounds. (B) Control cells were cultured with compounds indicated in black letters. WNR refers to Wnt3a, Noggin and RSPO1, and was abbreviated to hICs cultured with these additions. Images were taken on days 10, 16, and 22. (C) TEER was measured every day starting 4 days after the 2D culture. Warmed medium was added to the insert at the time of measurement. Mean±s.d., *n*=3.

**Fig. 7.**
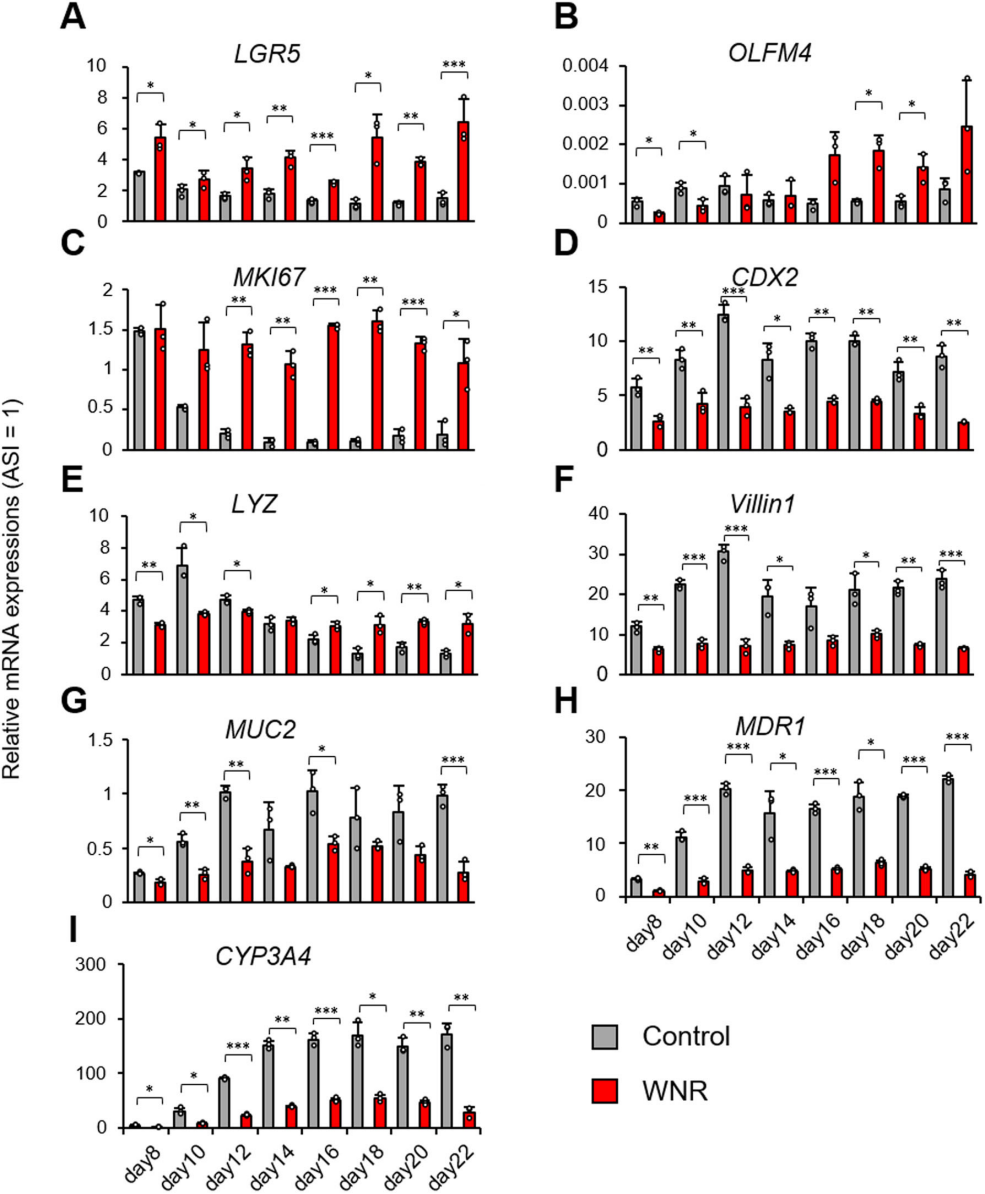
**WNR contributes to the maintenance of the degree of hIC differentiation.** The expression levels of mRNA for intestinal-related genes were measured via qPCR. The delta–delta method was used, and the data were normalized by HPRT. The relative values obtained from the adult small intestine (ASI) as 1 are shown. White dots: individual values; bars: mean±s.d. *n*=3. ^∗^*P*<0.05. ^∗∗^*P*<0.01. ^∗∗∗^*P*<0.001. (A-E) Genes contributing to the maintenance of intestinal stem cells. (F-I) Genes encoding mature intestinal markers and pharmacokinetic functions.

The previous culture results were recorded up to day 10. However, we further evaluated the properties of the cells by culturing them for a longer period of up to 22 days (Fig. 6A). In the late stage of culture with Wnt3a, Noggin, and RSPO1 (WNR), the amount of brown haze was reduced after washing the cells with the medium; these were likely stacked dead cells. WNR was used as a culture factor for maintenance. This reportedly contributes to stem cell maintenance during intestinal organoid culture, and the addition of WNR at appropriate time points was effective (Fig. 6B). TEER was stabilized at 150 Ωcm^2^ by the addition of WNR, thereby suppressing the excessive increase (Fig. 6C). Gene expression analysis revealed that, in the WNR culture, significantly higher expression levels of the intestinal stem cell markers *LGR5* and *OLFM4* and the transient amplifying (TA) cell marker *MKI67* were maintained (Fig. 7A–C). Similarly, the expression of *LYZ*, a Paneth cell marker responsible for the intestinal stem cell niche, was also maintained. The expression of *CDX2*, a midgut marker, was higher in the WNR culture than in the adult small intestine, but it was also higher in the control (Fig. 7D,E). In contrast, the expression levels of the genes *villin1* and *MUC2* and the pharmacokinetic-related markers *CYP3A4* and *MDR1* were elevated in the control, but relatively high expression levels were maintained in the WNR culture (Fig. 7F–I).

## DISCUSSION

In this study, we successfully induced the differentiation of intestinal cells that possess structures similar to those of cells in the living intestinal tract. The resulting cells be used as a high-throughput screening (HTS) evaluation system by culturing intestinal organoids in a 2D ALI culture system. Although several studies have induced hIC differentiation in a 2D culture system, most cells had a monolayer structure consisting of absorptive epithelial cells (Iwao et al., 2014 et al, 2015; Kodama et al., 2016; Kabeya et al., 2018; Ogaki et al., 2013; Negoro et al., 2018); these are not suitable for evaluating complex cell–cell interactions, interactions with intestinal bacteria, and inflammatory responses. Meanwhile, intestinal organoids contain various cell types that comprise the intestinal tract and might be similar in nature to living organisms (Spence et al., 2011; Uchida et al., 2017; Finkbeiner and Spence 2013). However, their characteristic morphology with luminal structures is unsuitable for use in HTS and quantitative evaluation. Conversely, the intestinal cells produced in this study are a stable supply for the HTS evaluation systems, because instead of a suspension culture, a 2D culture system is used, which allows for mass culture. Moreover, because many intestinal organoids are used for the 2D culture, individual differences in intestinal organoids are eliminated and more homogeneous cells can be cultured. These intestinal cells can be used as an evaluation system in drug discovery research, enable complex evaluations that were not previously possible.

Crypt-villus-like structures formed in the ALI culture with activating factors for both Wnt and cAMP signalling. CHIR99021, a factor that enhances Wnt signalling, and A-83-01, an inhibitor of TGFβ signalling, are important for the maintenance and culture of intestinal stem cells (Kondo et al., 2020). Furthermore, cAMP signalling in intestinal cells upregulates CDX2 expression, a nuclear transcription factor involved in the formation of the gastrointestinal tract (Suh and Traber, 1996; Chen et al., 2005; Coskun et al., 2011). In this experiment, the medium containing factors such as forskolin, CHIR99021, and A-83-01 was supplied only from the basement membrane side in order to maintain intestinal stem cells on that side. This may have caused the formation of a crypt-like structure in the lower part and a villus-like structure in the upper part of the intestine. Notably, CHIR99021 is presumed to play a very important role in the maintenance of intestinal stem cells because of the experimental results of hICs. TEER and mRNA expression analysis suggested that early-stage organoids were superior to HIOs used for hIC culture, especially in terms of cell proliferation capacity and TEER. This suggests that intestinal stem cells, which are abundant in the early stages of HIO culture, are important in the formation of the 3D structure of hICs. In fact, the TEER was maintained at a high level after the addition of CHIR99021, which is useful for the maintenance of intestinal stem cells, suggesting that even significantly aged intestinal stem cells can be maintained by CHIR99021 and ALI. In addition, this also improved the expression of various intestinal-related genes, suggesting a positive impact on the induction of differentiation. It is also believed that mature HIOs have stronger intercellular adhesion and incur more damage during cell detachment, which is related to the poor condition of hICs. Two types of coating agents, iMatrix-511 silk and Matrigel, were used for comparison. In living organisms, the extracellular matrix, which constitutes the basement membrane, is essential for cell maturation and homeostasis. One major component of the extracellular matrix is laminin, which has several subtypes. Since the basement membrane of the intestinal tract contains a large amount of laminin α5, we believed that cell culture plates coated with iMatrix-511 silk, a fragment of laminin α5, could be used to culture cells in an environment similar to that *in vivo* (Mahoney et al., 2008). Our analysis revealed that iMatrix-511 silk coating tended to produce higher TEER values, but these values were almost similar to those obtained with Matrigel. This suggests that Matrigel contains laminin and that the other inclusions exerted no significant effect on the cells. In addition, the cells could be preserved in several commercially available cell preservation solutions, making this a strong advantage from a convenience standpoint.

The generated hICs demonstrated well-developed microvilli on the cell surface, and the expression and secretion of mucin was also evident on immunofluorescence staining. The fluorescence images also clearly showed that the hICs were not flat structures but rather, uneven 3D structures. Cell3iMager Estier was used to analyse the 3D structure over time and observe the cell morphology during culture. A very large difference was noted between the normal culture and ALI culture. First, cells in the normal culture had a low height and flat structure, whereas those in the ALI culture had an uneven structure throughout (similar to villi). These structural changes were noted around day 6, and the crypt-villus-like 3D structure was formed over approximately 2 days. Thereafter, the height of the cells remained constant until day 10. Although the factors that may contribute to the formation of the villus-like structure were described earlier, CHIR99021, which enhances Wnt signalling and is the most important signal for the maintenance of intestinal stem cells, was added only up to day 4. Nevertheless, the morphology of the cells changed significantly after day 6, suggesting that ALI culture does not directly promote the formation of the 3D structure, but rather indirectly promotes it via other factors such as cell proliferation. Forskolin and factors that activate cAMP signalling (i.e. 8-Br-cAMP and IBMX) promoted the formation of the crypt-villus-like structures, suggesting that the activation of cAMP signalling is essential for their formation. Intestinal cells in living organisms have a very rapid turnover; these migrate from the crypts to the apical end and are extruded into the lumen after approximately 7 days. Thus, the division speed of intestinal stem cells maintained by this culture method is balanced by rapid turnover of cells that are shed on the apical membrane side; this maintained a cell height of approximately 150 μm. In line with this, after day 8, a large number of dead cells accumulated on the cell surface. Furthermore, the height of the villi was approximately 150 μm, which was lower than that *in vivo*, possibly due to the proliferation rate of the cells and their short lifespan. Additionally, the apical side of cells had a straight and flat structure in the normal culture as seen via TEM, whereas an uneven structure was noted in the ALI culture. Microvilli and tight junctions, indicators of cell maturity, were also observed in both cultures. In particular, the microvilli in the ALI culture were approximately twice as long and more dense than those in the normal culture. Microvilli increase the surface area of the intestinal tract, which is advantageous for the absorption and excretion of drugs.

Intestinal cells cultured in 2D systems have a short lifespan and can rarely be used for long periods of time. However, they have crypt-villus-like structures and undergo a consistent turnover, continuously replacing old cells with new ones, as in living organisms. We attempted to maintain the culture by adding WNR and extending the addition period of CHIR99021, based on culture factors for intestinal organoids were maintained for a long period of time by a similar mechanism (Mussard et al., 2020; Kardia et al., 2021). Consequently, we succeeded in maintaining the cryptic villus-like structure until day 22, and the TEER value also did not increase compared to the control, suggesting that the culture was maintained under normal conditions. Gene expression analysis revealed the increased expression of intestinal stem cell markers, TA cell markers, and Paneth cell markers, whereas the expression of transporters and metabolic enzymes was decreased compared to the control. Conversely, the control group had significantly higher gene expression of *Villin1*, *MUC2*, and other genes, likely because all intestinal stem cells and immature cells were replaced by mature epithelial cells. Since the mRNA expression level of hICs is already sufficient at around day 10, we believe that WNR suppressed the excessive maturation of hICs and allowed them to maintain an appropriate balance with the intestinal stem cells.

These results are highly innovative in research of 2D culture of intestinal tract cells and intestinal organoids. We successfully cultured cells with complex shapes such as intestinal organoids on an extremely simple culture platform known as a cell culture insert, which also allows for long-term culture. These cells have highly similar characteristics to living intestinal tissues, and they can be used as a better evaluation system versus conventionally cultured cells of the intestinal tract. This method can enable a wide range of evaluations, such as those involving long-term drug toxicity evaluation and inflammation repair tests using therapeutic drugs. Although the detailed mechanism underlying the formation of the 3D structures and their pharmacokinetic functions requires further evaluation, we anticipate further developments in intestinal research through the use of these intestinal cells.

## MATERIALS AND METHODS

### Culture of iPS cells and inducing differentiation into HIOs

A human iPS cell line (Windy) was provided by Dr A. Umezawa of the National Center for Child Health and Development (Tokyo, Japan); this was cultured as previously reported (Onozato et al., 2021). The differentiation from iPS cells to HIOs was induced using methods based on previous reports, with some modifications. In short, iPS cells were exposed to activin A for 3 days to generate endoderm-like cells, and then these were exposed to FGF4 and CHIR99021 for 4 days to induce differentiation into hindgut-like cells. The hindgut-like cells were passaged onto laminin-511 E8 fragment-coated plates and cultured for 72–96 h to 90% confluence in medium containing 5% fetal bovine serum (FBS), EGF (100 μg ml^−1^), CHIR99021 (3 μM), A-83-01 (500 nM), Y-27632 (10 μM), and FGF2 (30 ng ml^−1^). The cells were once again passaged and cultured for 72–96 h. Afterward, they were seeded at a density of 5.0×10^6^ cells into EZSPHERE and cultured for 3 days to produce spheres. After sphere preparation, the cells were grown into HIOs via floating culture in 3% Matrigel-containing medium for 6–18 days.

### Single-cell formation and cryopreservation of HIOs by detachment

After inducing HIO differentiation, the HIOs were exposed to Y-27632 (10 μM) for 3 h. They were collected in a tube, centrifuged at 200* **g*** for 5 min, then washed with phosphate-buffered saline (PBS) (−). Next, they were treated with EDTA (5 mM) for 5 min and washed again with PBS (−). After removing PBS (−), TrypLE Select was added and incubated at 37°C for 15 min. Pipetting was performed every 5 min during incubation to accelerate cell detachment. After single-cell formation, 10% FBS-containing medium was added to stop the detachment reaction. Then, the cells were completely detached using a cell strainer and centrifuged at 200* **g*** for 5 min. For cryopreservation, the cells were suspended in a cryopreservation solution at the recommended cell number, then frozen using the slow freezing method. Commercially available cryopreservation solutions (CELLBANKER^®^ 1, CELLBANKER^®^ 1plus, STEM-CELLBANKER^®^ GMP grade, TC Protector, and StemSure^®^ Freezing Medium) were used.

### 2D culture of the HIOs

The inserts used in the experiment had a pore size of 0.4 μm and were coated with the laminin-511 E8 fragment. Single-celled HIOs were suspended in the culture medium and seeded into 24-well inserts at 1.3×10^5^ cells/well. The first medium change was done at the start of ALI culture (i.e. day 3 of culture). The culture medium contained advanced DMEM/F-12 supplemented with 2% FBS, 2% B27 supplement, 1% N2 supplement, 1% PS, 1% GlutaMax, and HEPES (15 mM). Various compounds were added according to the number of culture days, as shown in Figs 2A and 6A. The medium was changed once every 2 or 3 days. ALI culture was performed by removing the medium inside the insert and exposing it to air, and the medium seeping into the insert was removed once every 24 h to keep it medium-free. Furthermore, the dead cells stacked on the top of the cell sheet were washed off daily with the medium.

### Reverse transcription–quantitative polymerase chain reaction (RT-qPCR) analysis

The total RNA was extracted according to the manual accompanying the Agencourt RNAdvance Tissue Kit after cell collection. The amount of RNA was determined by measuring the absorbance at 260 nm using the NanoDrop One ultratrace spectrophotometer in the RNA simple quantification mode (Thermo Fisher Scientific, Waltham, MA, USA). RNA purity was determined using the ratio of absorbance at 260–280 nm. Meanwhile, cDNA synthesis was performed using the ReverTra Ace^®^ qPCR RT kit according to the manufacturer's instructions. The PCR primers are listed in Table 1. The qPCR mixtures for RT-qPCR were prepared using the KAPA SYBR Fast qPCR kit with a final volume of 10 μl. Reactions were performed on a LightCycler^®^ 96 System (Roche Diagnostics, Japan) with initial denaturation at 95°C for 3 min, followed by denaturation at 95°C for 3 s, then annealing and extension reactions at 60°C for 31 s, done for 40 cycles. The results were corrected using *hypoxanthine phosphoribosyltransferase 1* (*HPRT*) as an intrinsic control.

**Table 1.** Gene sequences of the primers

Gene name	Sense (5′-3′)	Antisense (3′-5′)
*CDX2*	ACCTGTGCGAGTGGATGC	TCCTTTGCTCTGCGGTTCT
*CYP3A4*	CTGTGTGTTTCCAAGAGAAGTTAC	TGCATCAATTTCCTCCTGCAG
*HPRT*	CTTTGCTTTCCTTGGTCAGG	TCAAGGGCATATCCTACAACA
*LGR5*	TGCTCTTCACCAACTGCATC	CTCAGGCTCACCAGATCCTC
*Lysozyme*	TCAATAGCCGCTACTGGTGT	AATGCCTTGTGGATCACGGA
*MDR1*	CCCATCATTGCAATAGCAGG	TGTTCAAACTTCTGCTCCTGA
*MUC2*	AGAAGGCACCGTATATGACGAC	CAGCGTTACAGACACACTGCTC
*OLFM4*	CAGACACCACCTTTCCCGTG	CCTTCTCCATGATGTCAATTCGG
*Villin1*	AGCCAGATCACTGCTGAGGT	TGGACAGGTGTTCCTCCTTC

### Immunofluorescence staining

After differentiation, the hICs were fixed with 4% paraformaldehyde. Then, these were permeabilised with 10% Triton-X-100 and blocked in phosphate-buffered saline (PBS) containing 1 mg ml^−1^ BSA for 30 min, then treated with the primary antibodies at 4°C overnight. After rinsing, the sections were incubated with secondary antibodies at room temperature for 1 h. Nuclei were stained using Hoechst33342. The antibodies and their dilutions are summarised in Table 2. After staining, the bottom of the insert was cut out and placed on a microscope slide with the seal (Geneframe, Thermo Fisher Scientific), mounted using ProLong Diamond Aitifade Mountants and fixed with a cover slip, then captured and analysed using an ECLIPSE Ti2 inverted microscope (Nikon, Tokyo, Japan).

**Table 2.** Antibodies and other substances for immunofluorescence staining

Name	Host	Source	Catalogue number	Dilution
Anti-MUC2	Mouse	Abcam	ab11197	1:200
Anti-mouse 555	Goat	ThermoFisher	A32727	1:500
Hoechst33342	-	Dojindo	H342	1:500
Alexa Fluor 488 Phalloidin	-	ThermoFisher	A12379	1:40

### Observation of the 3D structure of cells using Cell3iMager Estier

After day 4 of culture, specific positions in each well were imaged every 12 h. During imaging, a small amount of medium was added to suppress light reflection, and a simple incubator was used to minimise damage to the cells. The images were analysed using ImageJ. The membrane portion of the cell culture insert was removed, then the cell portion was extracted. Afterward, heat mapping and searching for the maxima were performed.

### Transmission electron microscopy

The hICs were fixed overnight with 2.5% glutaraldehyde at 4°C, then postfixed with 1% osmium tetroxide for 2 h at 4°C. The samples were dehydrated with ethanol, embedded in resin, and cut into 0.1-mm sections. These sections were stained with uranyl acetate and observed using a Hitachi H7600 transmission electron microscope (JEOL, Tokyo, Japan).

### Statistical analysis

Statistical significance was evaluated using the Games–Howell test for multiple comparisons.

## Supplementary Material

10.1242/biolopen.061612_sup1 Supplementary information
