# Supplementary material for: Air–liquid interface culture combined with differentiation factors reproducing intestinal cell structure formation in vitro
Source: Biol Open. 2025 Jan 20;14(1):bio061612. doi: 10.1242/bio.061612 (PMC11789277; doi:10.1242/bio.061612)
Supplement: Supplementary information [file biolopen-14-061612-s1.pdf]

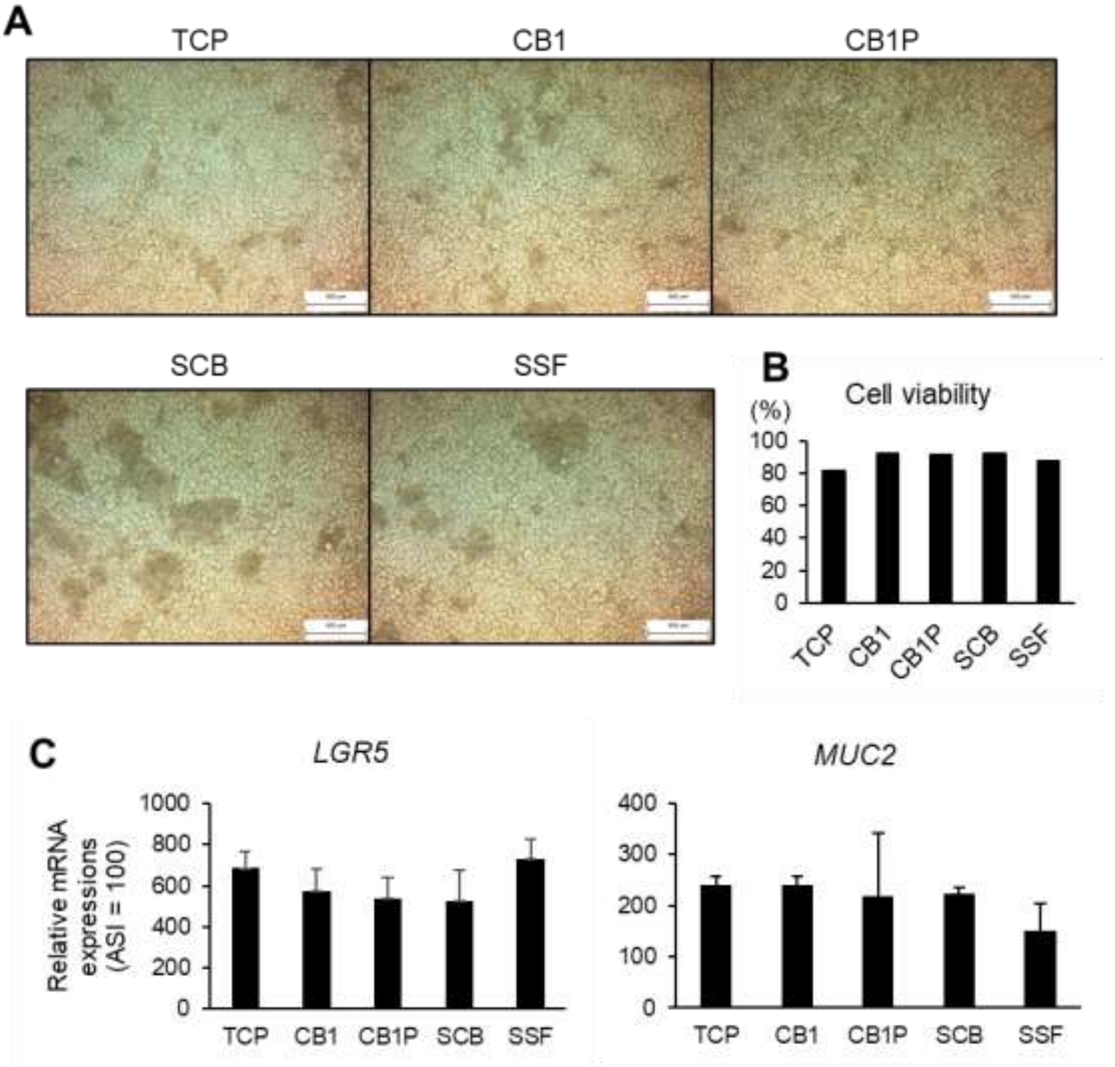

Fig. S1. hICs can be cryopreserved by slow freezing. (A) The HIOs were dispersed to single-cell and then stored in five different cryopreservation solutions. The types were CELLBANKER® 1 (CB1), CELLBANKER® 1plus (CB1P), STEM-CELLBANKER® GMP grade (SCB), TC Protector (TCP), and StemSure® Freezing Medium (SSF). After storage in liquid nitrogen for at least one month After storage in liquid nitrogen for at least one month, cells were seeded and cultured. Cell morphology was photographed on day 10 of culture. Scale bars = 100 µm. (B) The percentage of cells alive when thawed relative to when cells were stored. Mean, n = 1. (C) Expression levels of mRNA were measured by qPCR. The delta-delta method was used, and data were normalized by *HPRT*. Relative values obtained from adult small intestine (ASI) as 100 are shown. Mean + S.D. n = 3.

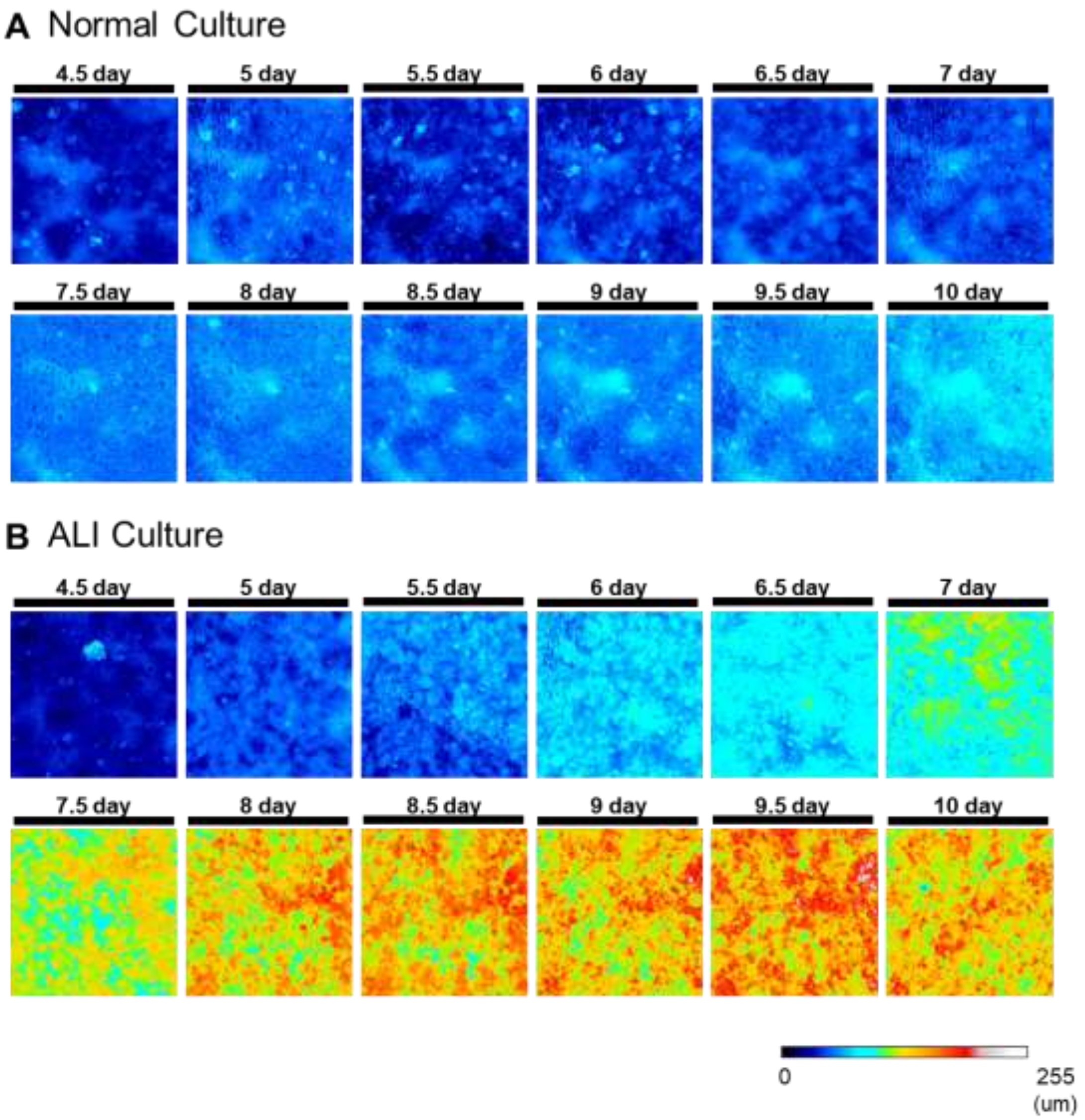

Fig. S2. ALI thickens the cell three-dimensionally. hICs were cultured in Normal and ALI culture methods for 10 days. The same position was imaged every 12 hours using Cell3iMager Estier. The images were processed by ImajeJ to create a heat map to visualize the three-dimensional structure. Before imaging, dead cells accumulated on the hIC were washed away, and 50 μL of medium was added to the insert to prevent diffuse reflection of light before imaging.

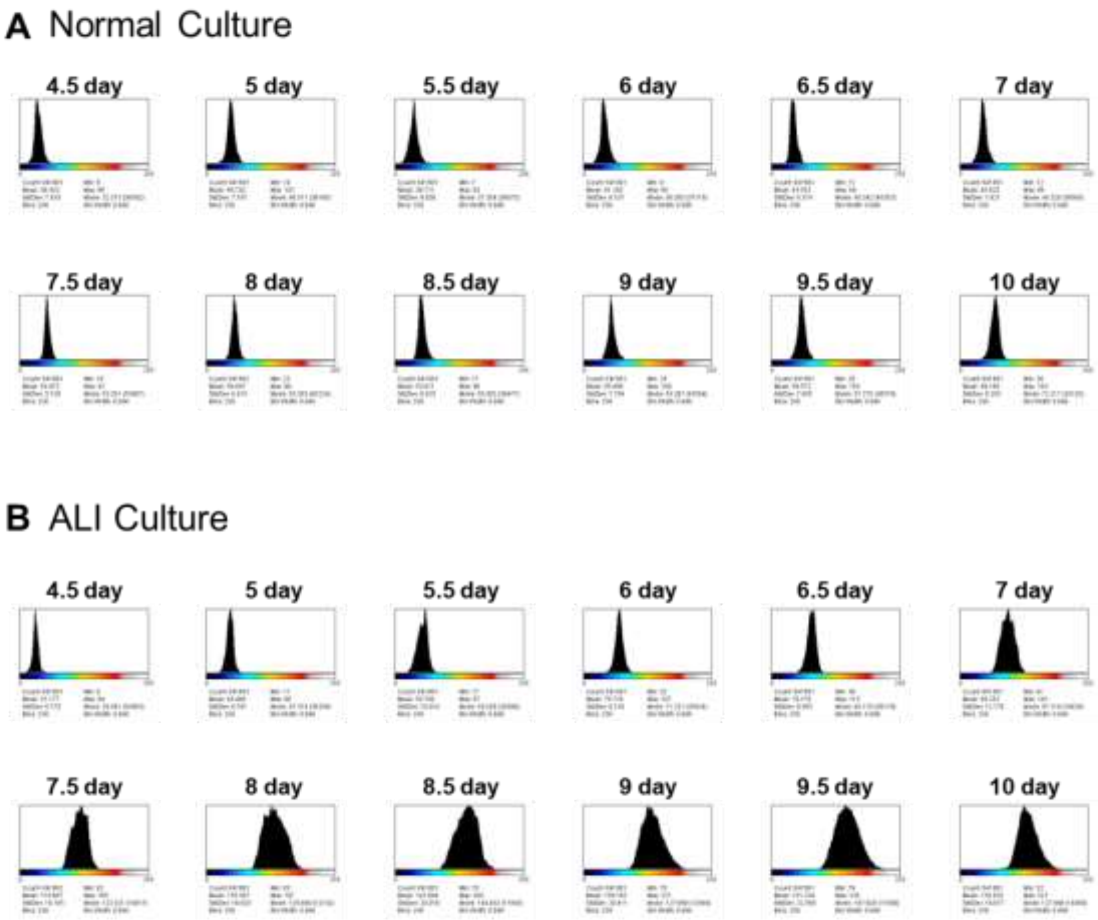

Fig. S3. ALI causes variation in cell thickness. To make it easier to understand the thickness variation of the hICs, histograms were created. Since the same imaging datas were used as in Figure 12, the heatmap of Figure 12 was converted to a histogram. The thickness was quantified by ImajeJ.

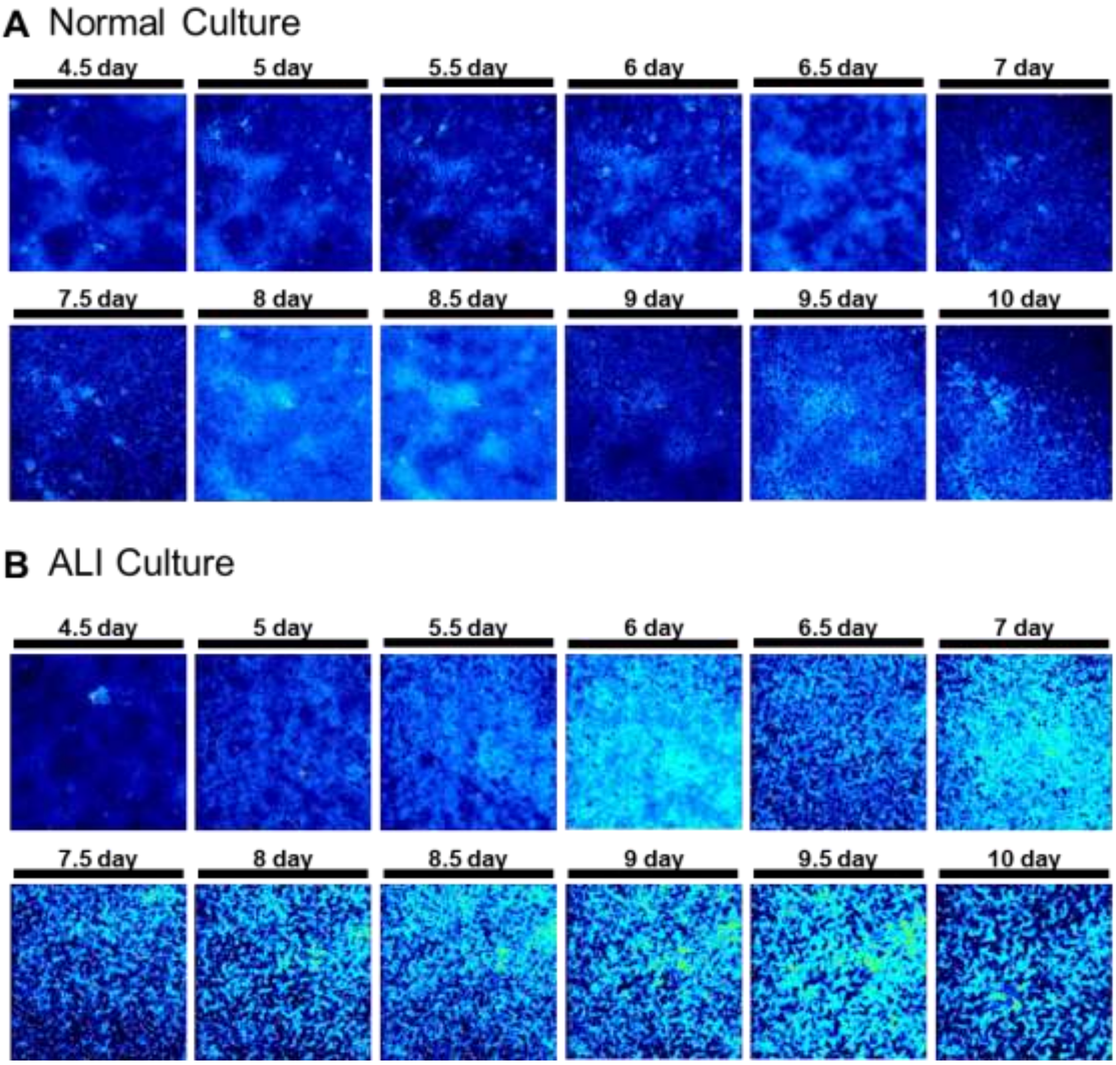

Fig. S4. ALI culture creates a convex structure. We observed how the morphology of hIC changed over time. A Cell3iMager Estier was used for imaging, and the same locations were imaged every 12 hours until day 10 of culture. Dead cells on the hIC were removed prior to imaging to allow clear observation of the structure, and the maxima of the villi were marked by ImajeJ. White dots indicate convexity.

Biology Open • Supplementary information

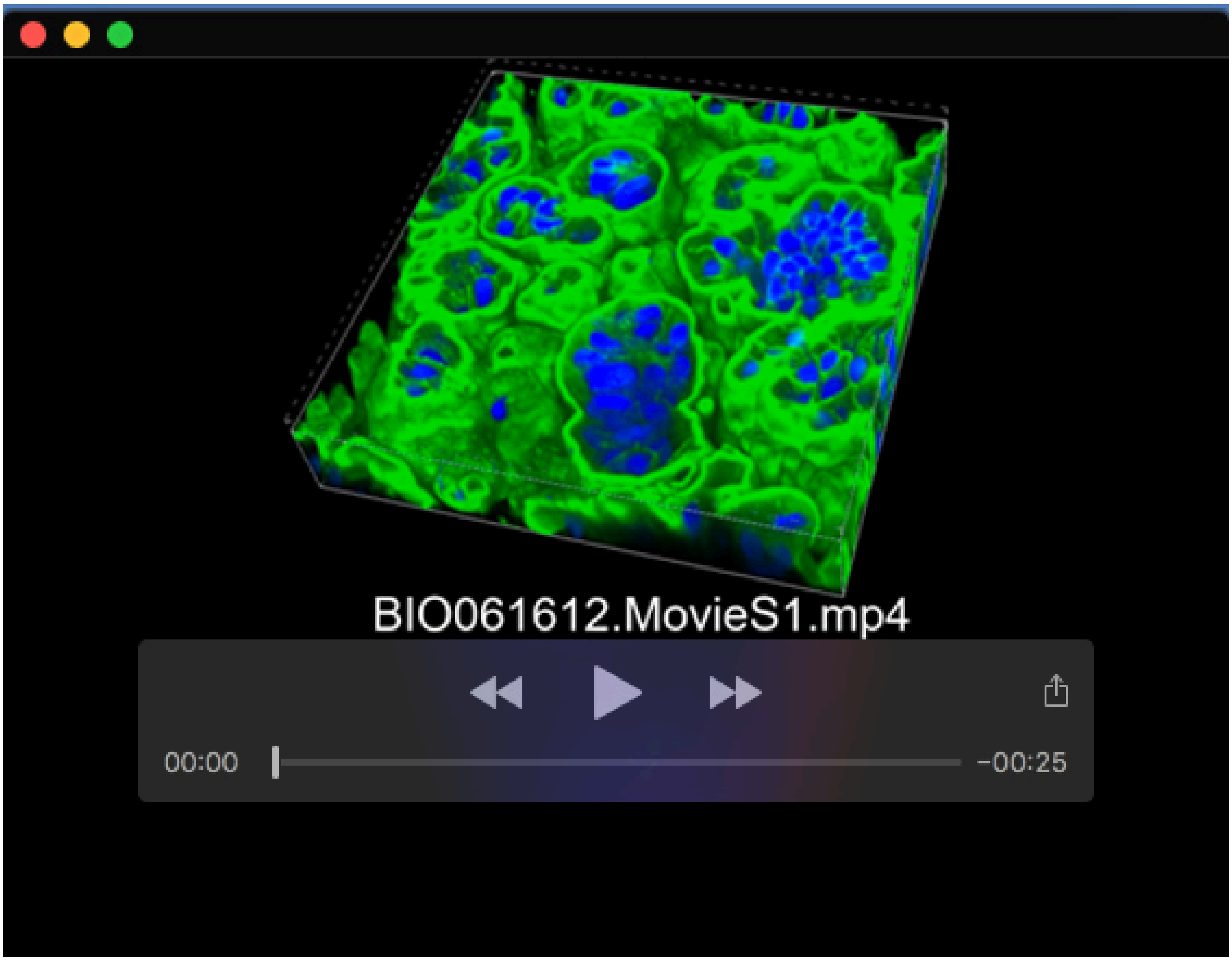

Movie 1. Microvilli expression and three-dimensional structure of hICs. The images taken in Figure 4 were superimposed in the Z-axis direction to produce a 3D view. 0.19 µm widths were taken for a total of 110 µm.
